# HipScreen: a valid mobile app to measure hip migration in children with cerebral palsy in the community setting

**DOI:** 10.1302/2633-1462.74.BJO-2025-0322.R1

**Published:** 2026-04-15

**Authors:** Akib M. Khan, John Amen, Oliver Perkins, Konstantinos Kafchitsas, Stephen J. Cooke, Michail Kokkinakis, HipScreen Study collaborators

**Affiliations:** 1 Department of Paediatric Orthopaedic Surgery, Chelsea and Westminster Hospital NHS Foundation Trust, London, UK; 2 Division of Paediatric Orthopaedics, Department of Orthopaedic Surgery, Ain Shams University, Cairo, Egypt; 3 Department of Paediatric Orthopaedic Surgery, Evelina London Children’s Hospital, Guy’s and St Thomas’ NHS Foundation Trust, London, UK; 4 Department of Orthopaedics and Traumatology, University of Patras, Patras, Greece; 5 Department of Paediatric Orthopaedic Surgery, University Hospitals Coventry and Warwickshire NHS Trust, Coventry, UK; 6 Department of Paediatric Orthopaedic Surgery, Evelina London Children’s Hospital, Guy’s and St Thomas’ NHS Foundation Trust, London, UK; 7 King’s College London, London, UK; 1 University Hospitals Coventry & Warwickshire NHS Trust, Coventry, UK; 2 Royal Wolverhampton NHS Trust, Wolverhampton, UK; 3 Staffordshire and Stoke On Trent NHS Partnership Trust, Stoke On Trent, UK; 4 Physio CYP, Birmingham, UK; 5 University Hospitals Coventry & Warwickshire NHS Trust, Coventry, UK; 6 Evelina London Children’s Hospital, London, UK; 7 Queen Mary’s Hospital, Sidcup, London, UK; 8 Rainbow Centre, East Kent University Foundation Trust, London, UK

**Keywords:** Cerebral palsy, CP, Hip migration, Hip dislocation, hips, radiographs, intraclass correlation coefficient (ICC), physiotherapists, paediatric orthopaedic, Hip displacement, paediatric orthopaedic surgeon, paediatricians, Pearson correlation coefficient

## Abstract

**Aims:**

The Reimers migration percentage (MP) is the gold-standard radiological parameter used to aid decision-making regarding surgical management of hip displacement in cerebral palsy (CP). Accurate measurement is important to risk stratify patients and allow timely onward referral from community teams to paediatric orthopaedic services. We performed a study to determine whether experts and novices could use a free smartphone app (HipScreen (HS) app) as a valid method for measuring MP in CP.

**Methods:**

Using the HS app, two groups measured MP in 20 pelvis radiographs (40 hips) at weeks 0 and 2. The ‘inexperienced’ group included four community physiotherapists with no CP experience. The ‘experienced’ group included four community physiotherapists and two paediatricians with CP experience. All participants watched the tutorial videos on the HS app website. HS app measurements were then compared with gold-standard picture archiving and communication system (PACS) measurements conducted by a senior paediatric orthopaedic surgeon. Modified Pearson correlation (r) was used to determine inter-rater reliability between HS app and PACS measurements. Intraclass correlation coefficient (ICC) was then used to assess intrarater reliability. The mean absolute deviation (MAD) was calculated to compare raters with the gold standard.

**Results:**

HS app measurements in the experienced and inexperienced groups showed highly significant correlation with the gold-standard measurements (p < 0.001). There were no significant differences between intra- or inter-group measurements at weeks 0 and 2 with *r* > 0.86 and p < 0.001. Both inter- and intra-rater reliability were excellent with ICC > 0.9. There was no statistically significant difference in the MAD within individual measurements and compared with the gold standard.

**Conclusion:**

The HS app is accurate in measuring MP in CP when used by non-specialists and specialists. Non-specialists do not require additional supplementary training. These findings suggest app efficacy and safety in regional and national hip surveillance programmes for children with CP.

Cite this article: *Bone Jt Open* 2026;7(4):540–548.

## Introduction

Cerebral palsy (CP) describes a constellation of conditions related to a non-progressive insult to the developing brain causing disordered movement or posture. Hip displacement is a common musculoskeletal problem in CP, more prevalent in nonambulant children with higher levels of neurological and functional impairment, with 90.5% of those with a Gross Motor Function Classification System (GMFCS) V affected.^1^

The natural history of hip dysplasia in CP has been studied extensively, and the disease-specific validated Child Health Index of Life with Disabilities (CPCHILD) shows a significant reduction in health-related quality of life in children with CP who have dislocated hips.^3^ Early identification of children who are ‘at risk’ allows a ‘proactive’ rather than ‘reactive’ approach to hip displacement, with earlier intervention associated with improved CPCHILD scores.^4^ Therefore, evidence-based validated surveillance tools for hip dysplasia are important in improving care for CP patients. Several hip surveillance programmes have been developed which use radiological assessments to identify ‘at risk’ hips.^5,6^ Hip surveillance programmes rely on clinical and radiological assessments at appropriate intervals and frequencies as determined by the relative risk of hip displacement per GMFCS level.

The migration percentage (MP) is the gold-standard radiological measure of hip displacement, which has been extensively studied as a valid and reliable quantitative measure.^7,8^ Prerequisites for effective use of MP in hip surveillance include the use of radiographs of acceptable quality interpreted by qualified professionals and means for onward referral at set thresholds. Referral thresholds are not standardized, however MP thresholds between > 30% and 40% usually warrant referral to orthopaedic services.^9^ MP measurements are conducted by clinicians who are trained to do so, including orthopaedic surgeons, specialist physiotherapists, and/or radiologists.^7^ Access to this group is variable depending on local service provisions, with the potential for delays in interpretation and referral. In many healthcare settings, there is limited access to these professionals, which has created the need for validated tools to screen patients with this condition.

**Fig. 1:**
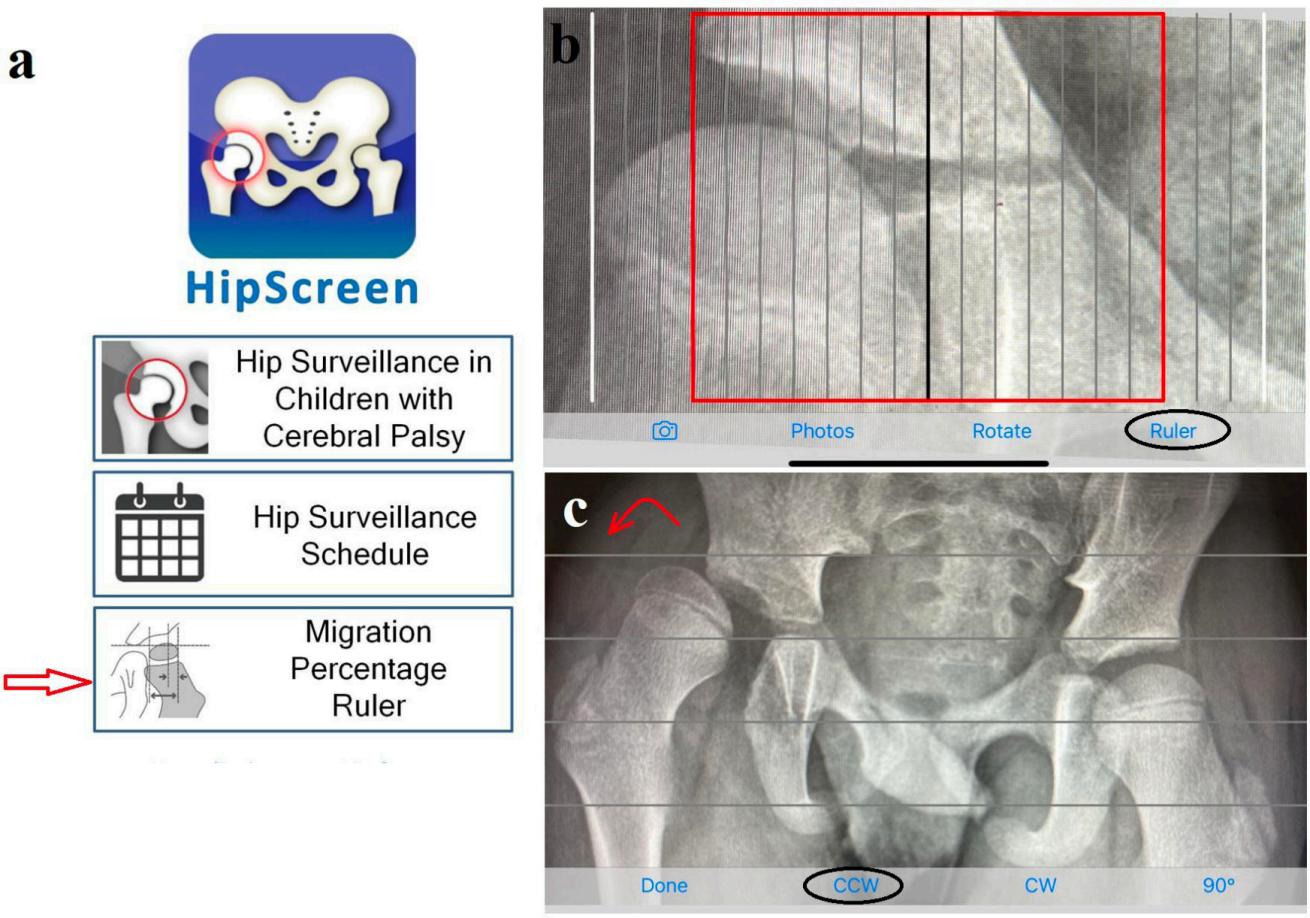
a) Home screen of the HipScreen (HS) app with a red arrow pointing to the ruler function. b) Ruler with the red line indicating 30% migration percentage. c) Rotate function to correct pelvic obliquity; in this radiograph, counter clockwise (CCW) is needed (red curved arrow). Permission obtained from original authors.^12^

The HipScreen (HS) app (Shriner’s Hospital for Children, USA) has been validated for use in hip surveillance of patients with hip subluxation in CP.^10^ It is a free resource available for download on different smartphone models. The HS app allows measurement of MP using the classic method (lateral border of the acetabulum) rather than the modified method.^11^ The app divides the ossific nucleus into ten partitions at 10% intervals of the entire width of the ossific nucleus (Figure 1). Our previous study has examined its accuracy, reliability, and discriminatory ability in measuring migration percentage among healthcare workers with experience in treating patients with CP.^12^ We aimed to investigate whether the use of the HS app could be used by a broader range of community-based healthcare professionals. We also aimed to assess whether additional training was necessary for novice users to increase accuracy of measurements.

## Methods

Ethical approval for this study was sought and deemed unnecessary by the Institutional Review Board at the Evelina London Children’s Hospital. Sample size was calculated using PASS v. 15 (NCSS, USA) with the type-1 error (α) rate of 0.05 and a power of 80%. This determined that a total of 35 hips was required. We anticipated a dropout rate of 10% and therefore deemed the use of 40 hips for analysis to be adequate. We identified 100 consecutive standardized pelvic radiographs from our CP surveillance programme and selected every fifth radiograph for inclusion. Our CP surveillance programme database included children of any age, CP type, and functional GMFCS level. All of our hip radiographs were obtained under the supervision of a senior radiographer with no forward or backward pelvic tilt, and with femora in neutral position so as to reduce the risk of measurement error.^13,14^ We excluded any with a fully contained hip, completely dislocated hip, or previous hip surgery on either one or both sides. In total, 20 radiographs (40 hips) were used in this study. Of note, as the same methodology was used, these were the same standardized and anonymized pelvic radiographs from our previous study.^12^

Gold-standard measurements for each radiograph were conducted and set using picture archiving and communication system (PACS) measurements by the senior author (MK) (Table I). A separate paediatric orthopaedic consultant (SJC) also measured the MP using both PACS and HS app to prove the validity of the software and confirm gold-standard measurements. We believe our methodology of using two experienced surgeons in setting the gold-standard measurements is sound, and the use of PACS matches globally accepted practice.^14^

**Table I.** Gold-standard measurements for each radiograph conducted by the senior author.

Patient number	Right	Left
1	40	35
2	40	30
3	30	35
4	65	30
5	50	20
6	30	60
7	30	35
8	30	40
9	40	25
10	35	30
11	55	55
12	35	30
13	40	65
14	45	50
15	30	30
16	40	35
17	30	30
18	40	45
19	30	25
20	20	45

The anonymized radiographs were inserted into two PowerPoint presentations (PPT1 and PPT2; Microsoft, USA) in different random orders. Two groups of professionals were identified from two geographically diverse areas of the UK. The first ‘inexperienced’ group had no experience in managing children with CP while the other ‘experienced’ group were professionals who routinely treat CP patients. The inexperienced group was composed of four community physiotherapists and the experienced group of four community physiotherapists and two community paediatricians. All participants were directed to complete the free online training resources provided by the HS app developers on their website.^15^ After each participant confirmed they had completed the online training, they were asked to complete MP measurements on PPT1. Following the first set of measurements, the inexperienced group were then given individual training sessions with the senior author (MK). All participants were asked to complete MP measurements on PPT2 two weeks later. All measurements were rounded to the nearest 5% which is in-keeping with the training material provided by the app developers.^16^

### Statistical analysis

Measurements from all participants were transcribed into table format and statistical analysis was performed using SPSS v. 27 (IBM, USA). The interobserver correlation was determined using the modified Pearson’s correlational coefficient (r), a reliability measurement between -1 (reverse reliability) and + 1 (perfect reliability). Measurements < 0.7 are considered unreliable, 0.7 to 0.8 moderately reliable, > 0.8 good reliability, and > 0.9 highly significant reliability. Significance calculations were also performed to determine a probability assessment of measurements. Intra-rater correlation was determined using the intraclass correlation coefficient (ICC). A score below 0.5 indicates poor reliability, 0.5 to 0.75 moderate reliability, 0.75 to 0.90 good reliability, and > 0.90 excellent reliability.^17^

Interobserver correlation in the experienced and inexperienced groups was calculated. Initially a normality test using the Kolmogorov-Smirnof test was conducted. In cases of normality, a paired *t*-test was performed and in case of not normal distributed data, a Wilcoxon-signed rank test was performed. A p-value < 0.05 was deemed statistically significant. In case of repeated comparisons (multiple null hypotheses with same data) the Holm-Bonferoni correction was also performed considering that this would otherwise increase the type-II error rate.

The mean absolute deviation (MAD) was calculated from the mean absolute difference taken between raters at week 0 and week 2. It was also calculated between the gold-standard measurement and the raters’ measurements at both week 0 and week 2. The SD for each dataset was also calculated.

## Results

### Interclass comparison

All HS app measurements showed high correlation with the gold-standard PACS measurements, with the Pearson coefficient r showing high correlation (> 0.9) in each measurement at both timepoints in the experienced (Table II) and inexperienced (Table III) groups. Further analysis of inter-rater reliability showed no single statistically significant measurement compared with the gold standard by any participant in either group at either timepoint (Table IV and Table V). There were only two statistically significant differences between experienced participants (experienced rater 5 vs rater 1 and 3 at the two-week measurements). However, all three raters were not statistically significant compared with the gold standard, and this finding is not thought to represent any clinical importance.

**Table II.** Validity assessment demonstrating interobserver correlation in measurements by the experienced group at week 0 and week 2, and comparison with the gold-standard picture archiving and communication system measurements.

r Wk 0	Exp1	Exp2	Exp3	Exp4	Exp5	Exp6	GS PACS
Exp1	1	0.934	0.918	0.930	0.910	0.890	0.917
Exp2		1	0.951	0.974	0.968	0.947	0.964
Exp3			1	0.953	0.941	0.896	0.926
Exp4				1	0.972	0.943	0.946
Exp5					1	0.948	0.948
Exp6						1	0.931
**r Wk 2**							
Exp1	1	0.975	0.962	0.955	0.941	0.932	0.959
Exp2		1	0.966	0.971	0.948	0.923	0.968
Exp3			1	0.954	0.915	0.908	0.953
Exp4				1	0.939	0.910	0.955
Exp5					1	0.902	0.933
Exp6						1	0.915

Exp, experienced practitioner; GS PACS, gold-standard picture archiving and communication system; r, Pearson’s correlation coefficient.

**Table III.** Validity assessment demonstrating interobserver correlation in measurements by the inexperienced group at week 0 and week 2, and comparison with the gold-standard picture archiving and communication system measurements.

r Wk 0	InExp1	InExp2	InExp3	InExp4	GS PACS
InExp1	1	0.93	0.856	0.924	0.960
InExp2		1	0.866	0.958	0.930
InExp3			1	0.891	0.860
InExp4				1	0.965
**r Wk 2**					
InExp1	1	0.950	0.956	0.961	0.970
InExp2		1	0.926	0.941	0.946
InExp3			1	0.928	0.940
InExp4				1	0.950

GS PACS, gold-standard picture archiving and communication system; InExp, inexperienced practitioner; r, Pearson’s correlation coefficient.

**Table IV.** Significance testing of interobserver correlation in measuring experienced group at week 0 and week 2, including comparison with the gold-standard picture archiving and communication system measurements.

p-value Wk 0	Exp1	Exp2	Exp3	Exp4	Exp5	Exp6	GS PACS
Exp1		0.170	0.443	0.200	0.064	0.160	0.266
Exp2			0.636	0.992	0.105	0.725	0.549
Exp3				0.650	0.280	0.536	0.805
Exp4					0.523	0.805	0.716
Exp5						0.696	0.716
Exp6							0.361
**p-value Wk 2**							
Exp1		0.513	0.942	0.578	0.025	0.305	0.866
Exp2			0.505	0.910	0.105	0.725	0.548
Exp3				0.566	0.027	0.281	0.925
Exp4					0.074	0.621	0.611
Exp5						0.201	0.300
Exp6							0.334

p-values calculated using Wilcoxon-signed rank test.

Exp, experienced practitioner; GS PACS, gold-standard picture archiving and communication system.

**Table V.** Significance testing of interobserver correlation in measuring inexperienced group at week 0 and week 2, including comparison with the gold-standard picture archiving and communication system measurements.

p-value Wk 0	InExp1	InExp2	InExp3	InExp4	GS PACS
InExp1		0.511	0.154	0.595	0.613
InExp2			0.385	0.880	0.821
InExp3				0.284	0.316
InExp4					0.855
**p-value Wk 2**					
InExp1		0.151	0.138	0.831	0.847
InExp2			0.950	0.098	0.124
InExp3				0.097	0.142
InExp4					0.854

p-values calculated using Wilcoxon-signed rank test.

GS PACS, gold-standard picture archiving and communication system; InExp, inexperienced practitioner; p, p-value; Wk, Week.

Analysis of the MAD using a paired *t*-test demonstrated no statistically significant difference between the inexperienced and experienced raters (p = 0.399, 95% CI -0.326 to 0.758). Overall, these findings validate the interclass reliability of the HS app in accurately determining MP in CP in inexperienced and experienced healthcare workers over time.

### Intraclass comparison

ICC showed excellent correlation (ICC > 0.9) in both groups (Table VI and Table VII). The MAD showed no statistically significant difference between week zero calculations and the gold standard (p = 0.525, 95% CI -0.227 to 0.420) and week 2 calculations and the gold standard (p = 0.626, 95% CI -0.170 to 0.271) (Table VIII).

**Table VI.** Intraclass correlation between week 0 and week 2 in the experienced group.

ICC	Exp1	Exp2	Exp3	Exp4	Exp5	Exp6
Exp1	0.972					
Exp2		0.986				
Exp3			0.971			
Exp4				0.985		
Exp5					0.969	
Exp6						0.954

Exp, experienced practitioner; ICC, intraclass correlation coefficient.

**Table VII.** Intraclass correlation between week 0 and week 2 in the inexperienced group.

ICC	InExp1	InExp2	InExp3	InExp4
InExp1	0.960			
InExp2		0.968		
InExp3			0.909	
InExp4				0.982

ICC, intraclass correlation coefficient; InExp, inexperienced practitioner.

**Table VIII.** Mean absolute deviation between measurements taken at week 0 and week 2 as compared with the gold-standard measurements.

Group member and measurement	MAD (%)	SD (%)
**Exp1**		
Week 0 vs Week 2	2.525	2.341
Week 0 vs gold standard	2.969	2.438
Week 2 vs gold standard	3.000	1.432
**Exp2**		
Week 0 vs Week 2	3.019	1.475
Week 0 vs gold standard	2.844	2.183
Week 2 vs gold standard	2.625	0.791
**Exp3**		
Week 0 vs Week 2	3.281	1.631
Week 0 vs gold standard	2.625	0.791
Week 2 vs gold standard	2.719	1.631
**Exp4**		
Week 0 vs Week 2	1.750	0.975
Week 0 vs gold standard	2.125	0.813
Week 2 vs gold standard	2.303	1.144
**Exp5**		
Week 0 vs Week 2	3.000	2.181
Week 0 vs gold standard	2.625	1.312
Week 2 vs gold standard	2.881	2.705
**Exp6**		
Week 0 vs Week 2	1.938	1.497
Week 0 vs gold standard	2.500	1.013
Week 2 vs gold standard	2.500	1.013
**InExp1**		
Week 0 vs Week 2	2.291	1.439
Week 0 vs gold standard	2.469	1.630
Week 2 vs gold standard	2.548	1.548
**InExp2**		
Week 0 vs Week 2	2.366	2.531
Week 0 vs gold standard	2.805	2.710
Week 2 vs gold standard	2.080	1.339
**InExp3**		
Week 0 vs Week 2	1.883	1.098
Week 0 vs gold standard	2.025	1.154
Week 2 vs gold standard	3.125	1.550
**InExp4**		
Week 0 vs Week 2	2.938	4.008
Week 0 vs gold standard	2.774	4.016
Week 2 vs gold standard	2.731	1.086

Exp, experienced practitioner; InExp, inexperienced practitioner; MAD, mean absolute deviation.

### Effect of training for the inexperienced group

Further statistical testing was performed assessing changes in scores between week 0 and week 2 for the inexperienced raters (Table IX). This revealed improvements in scores for two of the four participants following the in-person training. However, it should be noted that all scores in week 0 were already significantly correlated with the gold standard, and this improvement is unlikely to be of any clinical significance.

**Table IX.** Comparison of week 0 and week 2 scores for inexperienced raters.

Rater	p-value*
InExp1	0.055
InExp2	0.001
InExp3	0.009
InExp4	0.429

* Wilcoxon-signed rank test.

InExp, inexperienced practitioner.

## Discussion

CP hip surveillance programmes first began in Sweden in 1994 with the Uppföljningsprogram för cerebral pares (CPUP), which demonstrated a clear reduction in hip displacement through repeated, standardized assessments.^18^ Subsequent programmes have achieved similar results including the Cerebral Palsy Integrated Pathway Scotland (CPIPS) and the Cerebral Palsy Integrated Pathway (CPIP) in England and Wales.^1^ The current challenges faced by hip surveillance programmes are multiple and summarized well in Hughes et al’s^19^ recent work. The HS app may play a role in addressing some of these, including reducing resource requirements (i.e. more inexperienced users can accurately determine MP), consistent reliability of measurements, and standardizing the expanded use of technology. Additionally, the ability to use this tool in the community allows improved equity of access to care across geographically diverse regions.

Different protocols exist for timing and adequacy of radiographs, experience of those reporting the imaging and subsequent referral pathways. Standardized and successful completion of each of these steps should be the goal with a view to reducing errors and improving equitable care. Unfortunately, the standard of service received by children can vary significantly even within well-established programmes in developed countries. For example, in England, regional health provision is determined by integrated care boards (ICBs) that commission care services for children. According to the NHS England Commissioning framework for children and young people with CP, as of 2025, “8 out of 42 ICBs do not have a provider overseeing the care for people with cerebral palsy.”^20^ Another example of variability in regional services can be seen in Canada, where reporting of MP by radiologists is as low as 20% in paediatric hospitals and 3% in community hospital settings, despite being used routinely by the surveillance programme coordinators.^21^ From a practical perspective, variation can also exist due to local and regional factors including appropriate transportation for heavily disabled children, location of clinical assessment, imaging facility provisions, access to tertiary care, and funding. From a financial perspective, the use of a validated app in the community can also be cost-effective for the health service by reducing transportation needs. Case studies have shown that the average yearly cost for families to attend tertiary clinic appointments is up to £3,500.^20^ Reducing this financial burden for families also minimizes time away from work and education for children and their families.

Currently, most radiographs are interpreted manually by end-users (such as physiotherapists or surgeons). However, even when manual measurements are conducted, differences can exist between clinicians. Faraj et al^22^ conducted a study with two clinicians measuring MP at time 0 and six weeks later. They found significant intrameasurer and intrasessional errors of up to 22.4% (with an absolute difference between successive measurements range of 0% to 23%). Inter-measurer absolute difference was as high as 26.5% which may be considered clinically unacceptable. It may be postulated that with more clinical experience, the accuracy of MP measurements may improve. However, Analan et al^23^ found that the seniority of clinicians had no bearing on the accuracy of measurements. Of note, their study involved the use of PACS systems and psychiatrists conducting the measurements. They found that both the novice and experienced measurers were highly accurate in comparison with the gold standard. Our study demonstrated a similar finding, in that both inexperienced and experienced users were as accurate in their measurements. Another question is whether measurers in different health professional roles (i.e. physiotherapist, paediatrician, surgeon) have significant differences in measurement results. Our findings have demonstrated no difference between the HS app measurements by health professionals in any of these roles. We have shown that the HS app is a standardized, user-friendly tool which, following simple online training, enables users to make accurate and repeatable measurements of MP regardless of user experience. Previous studies have shown the reliability of using technology in reducing variation and inconsistencies in interpreting MP calculations. Kulkarni et al^10^ (developers of the HS app) studied a diverse group of users analyzing the MP in 40 hips over two timepoints, and found the HS app to be accurate with a sensitivity of 94% and specificity of 66% with good reliability between timepoints (ICC = 0.83). These findings mirror our own, which showed accuracy with all users demonstrating no significant difference from the gold-standard measurements (p < 0.001) and excellent reliability between timepoints (ICC > 0.9).

The previous arm of this study investigated the role of healthcare professionals in a hospital setting using the HS app in measuring MP in CP.^12^ It involved training of each member of the healthcare team prior to conducting their first round of measurements. The study found good inter- and intrarater reliability in measurements across different medical and allied health specialties. Our current study adds to this, in that it examines the use of the HS app in a community setting across a wide geographical area, and with no didactic training prior to the first round of measurements, in both experienced and inexperienced healthcare practitioners. The only educational material mandated was the freely available resources on the HS app website. Our study found that the initial measurements at week 0 were accurate in both the experienced and inexperienced groups and, although specific training by a senior clinician improved accuracy, this was not thought to be clinically significant, suggesting that the existing training materials are effective.

The use of AI will bring the ability to automate analysis of CP hip radiographs through machine-learning (ML). An example of a system that shows excellent promise is BoneFinder (The University of Manchester, UK).^24^ The developers of the system used 1,650 pelvic radiographs in children with CP to compare BoneFinder with manual measurements made by five experts. They determined a MAD of 5.7% (SD 8.5%) with an excellent ICC of 0.91 for MP. This finding was mirrored by work conducted by Hughes et al,^25^ who used BoneFinder to interrogate 1,018 CP hips from a national database. They showed good ICC (0.80 (95% CI 0.65 to 0.87)) between its results and CPIPS measurements (entered manually by end-users).^25^ The reliability of the system was poorer when image artefact (such as a groin shield), aberrant anatomy, or radiographs with inadequate visualization of the lesser trochanter. Another ML model (SVM Model, Turkey) developed by Ertan Birsel et al^26^ also demonstrated excellent ICC (> 0.95) and an accuracy of > 90%. Of note, they excluded any radiograph that was not deemed to meet strict standardization criteria, which may explain the excellent results when compared with BoneFinder. While these newer ML methods hold promise, the role of manual measurements still exists, particularly in those who do not have access to newer technology, or in cases where artefact or anatomical variations are present.

Although we commend the HS app as a reliable tool for the diagnosis and accurate measurement of MP in children with CP, there are several limitations to our study. The HS app requires rounding of measurements to the closest 5%, which leads to results that may be of lower accuracy than standard PACS measurements (where increments of < 1% may be measured). However, our findings show that HS app measurements were comparable with the gold-standard PACS measurements as well as being reliable within the internal measurements between timepoints. This suggests that the 5% incremental cutoff is not of significance compared with using PACS measurements. In fact, Reimers^27^ reported a standard error of 10% for MP based on an estimate of errors that occur on the line to the nearest millimetre. Similarly, the Melbourne group also found rate of variability in expert measurements on a single radiograph to 5.8% of the true value.^8^ A pragmatic limitation of our study is the requirement of users to own a smartphone that can host the HS app. A large proportion of the clinical workforce in our healthcare system have smartphones, and therefore this consideration may be more significant in resource-poor settings. However, regardless of healthcare setting, there are also questions regarding data protection and ensuring clinicians are compliant with local standards when using a third-party application. We used screenshot images of PACS radiographs on PowerPoint slides, which is a relatively low fidelity method for displaying digital images. Although accessing higher-resolution images may be possible in hospital settings, this may not be the case in community care, and therefore lower-fidelity displays may in fact be more realistic. Additionally, the users did not trial the HS app in non-digitally image-based settings, therefore it is unknown how the HS app would perform with acetate films. Another assumption was that, although the inexperienced group of physiotherapists were not specifically trained to manage CP in their daily practice, they may have prior experience during their qualification.

In conclusion, accurate measurement of the MP is important in ensuring an effective CP Hip surveillance programme. Our study has shown that the use of the free HS app is reliable, accurate, and safe in the community setting with both allied health practitioners who have and have not previously had experience in treating children with CP. Those who have not had experience treating CP children had improvement of scores when individual expert training was provided, however we found that this is unlikely to be clinically significant, given excellent initial scores achieved (in-keeping with the gold standard). The efficacy of the free online training material in obtaining accurate readings is sustained over multiple weeks.

**Take home message**

- This study validated the free HipScreen app as an accurate method for measuring Reimers migration percentage in children with cerebral palsy (CP), comparing both experienced and inexperienced community practitioners against gold-standard measurements by a senior paediatric orthopaedic surgeon.

- The app showed excellent agreement with gold-standard measurements, with strong inter- and intrarater reliability and no significant differences between specialists and non-specialists, even without extra training. These findings support the app’s safe use in regional and national CP hip surveillance pathways, helping community teams identify hip displacement earlier and streamline timely referral to paediatric orthopaedic services.

## Data Availability

The data that support the findings for this study are available to other researchers from the corresponding author upon reasonable request.
